# Association of living arrangements with happiness attributes among older adults

**DOI:** 10.1186/s12877-021-02017-z

**Published:** 2021-02-04

**Authors:** Eun Jeong Hwang, In Ok Sim

**Affiliations:** 1 Department of Nursing, Sehan University, 1113 Samho-eup, Yeongam-gun, Jeollanam-do 58447 Republic of Korea; 2 Red Cross College of Nursing, Chung- Ang University, 84 Heukseok-ro, Dongjak-Gu, Seoul, 06974 Republic of Korea

**Keywords:** Living arrangements, Happiness, Older adult, Social activities, Socio-physical environment

## Abstract

**Background:**

In Korea, along with the rapid aging of the population, older adults’ living arrangements have changed in various ways. In particularly, the happiness of older adults living alone warrants attention because they are more vulnerable to unhappiness than those living with families are. This study reports on the level of happiness among older adults in Korean and examines the potential mediating roles of depressive symptoms, present health status, socio-physical environment, social support networks, and social activities, and happiness in three different living arrangements, older adults living alone, with their spouse, or with their family.

**Method:**

Data for this study were extracted from the secondary data from the 2017 Korean Community Health Survey, a non-experimental, cross-sectional survey conducted among Korean individuals that were aged 65 and above (*n* = 14,687). The chi-square test, one-way ANOVA, and Logistic regression were used to explore the related factors of happiness among the three groups.

**Results:**

Findings revealed a significant difference in the happiness index among older adults living alone (6.22 ± 2.11), older adults living with their spouse (6.76 ± 1.99), and older adults living with their family (6.46 ± 1.94) (F = 88.69, *p* < .001). As the result of logistic regression, older adults living alone (odds ratio (OR) = 0.75, 95% confidence interval (CI) = 0.57–0.99) and those living with their family (OR = 0.80, 95% CI = 0.65–0.99) demonstrated greater happiness as the frequency of contact with their family increased. Older adults living with their spouse indicated an increase in happiness when their contact with friends was higher (OR = 0.69, 95% CI = 0.56–0.84).

**Conclusion:**

It was recognized that factors influencing happiness differed according to older adults’ living arrangements, thus suggesting that older adults’ happiness could be facilitated through interventions that consider their circumstances, including living arrangements.

## Background

In South Korea, the proportion of people aged 65 years and older was 7.03% of the total population in 2000, thus making it an aging society [1]. Subsequently, in 2018, South Korea went on to become an aged society as the proportion of people aged 65 years and older became 14.76% of the total population. With this rapid aging rate, older adults’ living arrangements have changed in various ways. Specifically, the proportion of older adults living alone increased from 16% in 2000 to 19.10% in 2017. The number of older adults living alone is expected to more than double this figure by 2035 [2].

Happiness is a universal value sought by each individual, and everyone has the right to enjoy it. It has also been emphasized as an important indicator of health and is predicted by the individual’s sense of coherence [3]. In the past, growth-oriented societies considered income and happiness proportional to each other. Therefore, gross domestic product was used as an alternative measure of happiness. However, economic growth has little effect on happiness, as shown by the Easterlin Paradox [4]. Currently, people have become more interested in happiness as an outcome in itself. Therefore, the Organization for Economic Cooperation and Development [5] suggested the use of the “Better Life Index” system as an indicator of happiness. Considering the current context pertaining to aging populations, it is important to focus on older adults’ happiness because, as their proportion in the total population increases, the influence of their happiness on the overall happiness of the society will also increase. Therefore, it would be difficult to understand national happiness if older adults are unhappy.

In general, social support or social resources are necessary to lead a happy and healthy life. Social support is an important component of older adults’ happiness and health [6–9]. Among the sources of social support, the family is considered as the most important factor that influences older adults’ happiness [3, 6–10]. The previous studies report the important influence of contextual factors on associations between living arrangements and the happiness [3, 8], between living arrangements and health status [3, 10] of older individuals. Bai, Yang, and Knapp [6] found that the formal support system for solitary older adults without family support did not contribute to their life satisfaction. Further, Chiang and Lee [3] reported that family relationships were positively correlated with happiness, sense of coherence, and perceived health. Yu, Hou, and Miller [11] found that older adults living alone were also more likely to experience lower levels of social support and social cohesion than those living with others. Additionally, older adults living alone were more likely to report feeling sad, hopeless, and worthless than those living with others [11]. Further, Weissman and Russell [10] reported that older adults living with others had the poorest health; they had serious psychological distress and limitations in activities of daily living (ADLs) as compared with older adults living with their spouses. Summarizing these previous studies, it has been explained that older adults are likely to experience higher happiness when they receive physical care, emotional support, and financial assistance from their family, and when they are connected with community networks. These findings also suggest that older adults’ feelings of happiness differ according to their living arrangements.

Studies have examined concepts similar to happiness, including well-being [7], quality of life [9, 12–14], life satisfaction [6, 15, 16], subjective well-being [17], good life, and better life [5, 18]. Previous studies [14, 19, 20] have suggested various factors influencing older adults’ happiness. Van Leeuwen et al. [14] identified the following nine quality of life domains for older adults living at home: autonomy, role and activity, health perception, relationships, attitude and adaptation, emotional comfort, spirituality, home and neighborhood, and financial security. In the Madrid International Plan of Action on Ageing, the United Nations [19] recommended the following three methods for facilitating older adults’ contribution to the society through vibrant aging: focusing on older individuals and their development, advancing health and well-being into old age, and ensuring enabling and supportive environments. The World Health Organization (WHO) [20] presented a global age-friendly city guide for happy and active aging, which includes living environment, transportation, social participation, community support, and health services. In general, previous studies have identified the following factors associated with older adults’ happiness: depression, health status, socio-physical environment, social support networks, and social activities. Considering differences in the degree of subjective well-being of older adults according to social network types [17], it is suggested that strategies with diverse networks should be considered while developing methods to facilitate a healthy and happy life. Therefore, it is important to identify factors influencing older adults’ happiness according to their living arrangements and, accordingly, develop strategies to improve their happiness.

### Purpose

This study compared the associations between depressive symptoms, present health status, satisfaction with socio-physical environment, social support networks, and participation in social activities with happiness among older adults living alone, with their spouse, or with their family.

Ultimately, this study aimed to provide useful information for the development of happiness programs that consider older adults’ living arrangements.

## Methods

### Design

The study employed a non-experimental, cross-sectional research design.

### Data collection and procedures

The secondary data used in this study were extracted from the 2017 Community Health Survey conducted by the Korea Disease Control and Prevention Agency (KDCA) [21]. This survey instrument was developed with the participation of many professors, researchers, and administrators in related fields along with a vast amount of literature review. The government has been encouraged to analysis this data by researchers to develop a comprehensive and multidimensional policies to support national people. These data are available free of charge for research purposes. These nationwide annual surveys utilize the public health center network consisting of around 250 public health centers located in each municipality. National resident registration data and housing data were used as data to select the samples. The sample selection for the 2017 survey was based on the target population of adults aged 19 years and older. The sampling method of the survey uses a multi-stage cluster sampling to finally select an average of 900 subjects per community health center. Finally, all sampled households were included in the survey. In the data collection process, trained researchers visited the selected households and explained the purpose of and confidentiality measures used in the survey to the respondents. Subsequently, they collected data through one-on-one face-to-face answer with the subject using a computer assisted personal interviewing. The data for this survey were collected using self-report structured questionnaires. The survey period was from August 16 to October 31, 2017. For the present study, data pertaining to those aged 65 years and older were selected. As a result, 14,687 individuals were selected and subsequently divided into the following three groups based on their living arrangements: older adults living alone, those living with their spouse, and those living with their family. The classification criteria were as follows: one-person households were classified as older adults living alone, two-person households responding as couples were classified as older adults living with their spouse, and households with two or more members, which did not include couples, were classified as older adults living with their family.

### Dependent variable

The happiness index for older adults consisted of one item “all things considered, how happy are you in life these days?” It was rated on a 10-point Likert scale, with higher scores indicating greater happiness. Specifically, one point indicated the highest degree of being unhappy and 10 points represented the highest level of happiness. Therefore, individuals with five points or less were considered as unhappy, and those with six points or more were considered as happy. For conducting a binary logistic regression analysis, older adults were classified into the following two categories based on their happiness index results: unhappy (score1–5) and happy (score 6–10). The happiness index of the subjects of this study was averaged 6 points, and based on this, the happiness group and the unhappy group were classified.

### Independent variables

The independent variables included depressive symptoms, present health status, satisfaction with socio-physical environment, social support networks, and participation in social activities. The data for this survey were collected using self-report structured questionnaires. Details of the scales and reliability of the factors have been provided in subsequent sections.

#### Depressive symptoms

Depressive symptoms were assessed using the following nine items: “no interest in or fun at work,” “sinking feeling, depression, and hopelessness,” “difficulty falling asleep or sleeping too much,” “feeling tired,” “lack of appetite or overeating,” “considering oneself worthless and a harbinger of misery,” “difficulty concentrating on newspapers or television,” “nervousness, anxiety or too much wandering,” and “believing that death is preferable to living or experiencing thoughts about hurting oneself.” Each item was rated on a four-point Likert scale (1 = never, 2 = felt for several days, 3 = felt for over a week, 4 = felt almost every day), with higher scores indicating higher levels of depression. The Cronbach’s α of the depression instrument was 0.83 in the present study.

#### Present health status

Present health status was assessed using the following five items: athletic ability, self-management, daily activities, pain and discomfort, and anxiety. Each item was rated on a three-point scale (1 = bad, 2 = somewhat bad, and 3 = good), with higher scores indicating better levels of health. The Cronbach’s α of the health status instrument was 0.83 in the present study.

#### Satisfaction with socio-physical environment

Satisfaction with socio-physical environment was assessed using the following seven items: “trust in neighbors,” “help from neighbors,” “neighborhood safety level (disasters, traffic accidents, work accidents, crime),” “natural environment (air and water quality),” “life environment (electricity, water, sewage, garbage collection, athletic facilities),” “condition of public transportation (buses, taxis, trains, subway),” and “condition of medical facilities (hospitals, community health centers, oriental hospitals, pharmacies).” Each item was rated on a dichotomous scale (1 = dissatisfied, 2 = satisfied). The Kuder-Richardson Formula 20 value of the socio-physical environment instrument was 0.60.

#### Social support networks

Social support networks were assessed using the following three items: “contact with family (or relatives),” “contact with neighbors,” and “contact with friends.” Each item was rated on a six-point Likert scale (1 = less than once a month, 2 = once a month, 3 = two or three times a month, 4 = once a week, 5 = two or three times a week, 6 = four or more times a week).

#### Participation in social activities

Participation in social activities was assessed using the following four items: “religious activities,” “belonging to communities,” “leisure activities,” and “charity activities.” Each item was rated on a dichotomous scale (1 = non-participation, 2 = participation).

### Ethical considerations

The KDCA provides the data used in this study free of charge for research purposes after following certain official procedures and receiving approval. An investigator visited the subject’s house and provided an explanation of the purpose and process of the survey, and after obtaining the written consent of the subject, the survey proceeded. The Institutional Review Board of Sehan University (approval number SH-IRB 2019–43) granted permission to conduct the present study. The study adheres to the Strengthening the Reporting of Observational Studies in Epidemiology (STROBE) statement.

### Statistical analyses

Data were analyzed using SPSS version 21 for Windows. Inferential statistical analysis was conducted using the chi-square test and one-way ANOVA. Logistic regression was performed to determine the independent factors associated with happiness among older adults living alone, living with their spouse, and living with their family.

## Results

### Sample characteristics

The present sample comprised 14,687 older adults: 3059 (20.8%) living alone, 6644 (45.3%) living with their spouse, and 4984 (33.9%) living with their family (Table 1). There were significant differences in the sociodemographic characteristics of the three groups (*p* < .001). Among older adults living alone, 692 (4.7%) were male and 2367 (16.1%) were female. Among those living with their spouse, 3786 (25.8%) were male and 2858 (19.4%) were female. Among those living with their family, 1845 (12.6%) were male and 3139 (21.4%) were female. The average age was 74.86 ± 6.45 years, 72.47 ± 5.50 years, and 73.56 ± 6.75 years for older adults living alone, those living with their spouse, and those living with their family, respectively (*p* < .001). Although the educational category of elementary school graduates had the highest percentage in all three groups, there was a significant difference in educational level between the three groups (p < .001). Regarding marital status, 2360 older adults living alone were widowed and 2736 older adults living with their family were married, with these categories having the highest percentages in these two living arrangements (p < .001). Regarding employment status, in all three groups, the highest percentage was observed for unemployment. In all three groups, the highest percentage was observed for those not eligible for basic livelihood rights. Regarding the monthly average income, the highest proportion was 500,000 to 990,000 Korean won for older adults living alone, 1,000,000 to 1,990,000 Korean won for those living with their spouse, and over 2,000,000 Korean won for those living with their family (*p* < .001).

**Table 1:** Sample characteristics (*N* = 14,687)^a^

Characteristics	Living alone (***n*** = 3059)		Living with their spouse (***n*** = 6644)		Living with their family (***n*** = 4984)		χ^**2**^ or F	***p***
	***n***	%	***n***	%	***n***	%		
Gender								
Male	692	4.7	3786	25.8	1845	12.6	1120.77	< .001
Female	2367	16.1	2858	19.4	3139	21.4		
Age group (years)								
65–69	763	5.2	2365	16.1	1737	11.8	344.96	< .001
70–74	769	5.2	2026	13.8	1287	8.8		
75–79	796	5.4	1443	9.8	970	6.6		
> 80	731	5.0	810	5.5	990	6.8		
M ± SD	74.86 ± 6.45		72.47 ± 5.50		73.56 ± 6.75		163.04	< .001
Range	65–100		65–97		65–105			
Educational level								
None	552	3.8	383	2.6	681	4.6	661.31	< .001
Elementary school	1257	8.6	2039	13.9	1797	12.3		
Middle school	503	3.4	1471	10.0	977	6.7		
High school	490	3.3	1684	11.5	1013	6.9		
College or higher	246	1.7	1052	7.2	508	3.5		
Marital status								
Married	173	1.2	6644	45.3	2736	18.6	8632.93	< .001
Divorced	434	3.0	0	0.0	203	1.4		
Widowed	2360	16.1	0	0.0	2003	13.6		
Never married	83	0.6	0	0.0	35	0.2		
Employment status								
Unemployed	2381	16.2	4702	32.0	3752	25.6	61.74	< .001
Employed	678	4.6	1936	13.2	1231	8.4		
Eligibility for basic livelihood rights								
Yes	502	3.4	263	1.8	297	2.0	545.17	< .001
In the past	45	0.3	29	0.2	69	0.5		
No	2509	17.1	6352	43.3	4618	31.4		
Monthly income (10,000 won)^b^								
< 50	1011	6.9	521	3.6	169	1.2	4585.51	< .001
50–99	1328	9.1	2020	13.8	591	4.1		
100–199	481	3.3	2144	14.7	941	6.5		
≥ 200	221	1.5	1929	13.2	3230	22.1		

^a^ Missing data: educational level for older adults living alone (*n* = 11), living with their spouse (*n* = 15), living with family (*n* = 8); marital status for older adults living alone (*n* = 9), living with family (*n* = 7); employment status for older adults living with their spouse (*n* = 6), living with family (*n* = 1); basic livelihood rights for older adults living alone (n = 3); monthly income for older adults. ^b^ 1USD = approximately 1200 Korean won

### Comparison of variables between the three groups

The results of the comparisons between depressive symptoms, present health status, satisfaction with socio-physical environment, social support networks, participation in social activities, and happiness in older adults living alone, those living with their spouse, and those living with their family have been presented in Table 2 and Table 3. Significant differences between the three living arrangements groups were observed for happiness, depressive symptoms and present health status (*p* < .001). In terms of satisfaction with the socio-physical environment, the three groups differed significantly in terms of trust in neighbors (*p* = .001), help from neighbors (*p* < .001), neighborhood safety level (*p* = .009), condition of public transportation (*p* = .007), and condition of medical facilities (*p* = .002). Regarding social support networks, the three groups differed significantly in the frequency of contact with family, neighbors, and friends (*p* < .001). Finally, with reference to social activities, the three groups differed significantly in their participation in religious, belonging to communities, leisure, and charity activities (*p* < .001).

**Table 2:** Comparison of Depressive Symptoms, Present Health Status^a^

Characteristics	Categories	Living alone (n = 3059)		Living with their spouse (n = 6644)	Living with their family (n = 4984)			F	***p***
		***n***	%	***n***	%	***n***	%		
Happiness (M ± SD)		6.22 ± 2.11		6.76 ± 1.99	6.46 ± 1.94			88.69	< .001
Depressive symptoms (M ± SD)		12.48 ± 4.29		11.20 ± 3.30	11.71 ± 3.76			127.84	< .001
Present health status (M ± SD)		13.05 ± 2.01		13.70 ± 1.80	13.33 ± 1.99			132.09	< .001

^a^ No responses were excluded

**Table 3:** Comparison of Socio-Physical Environment Satisfaction, Social Support Networks, Social Activity Participation, and Happiness ^a^

Characteristics		Categories	Living alone (n = 3059)		Living with their spouse (n = 6644)		Living with their family (n = 4984)		χ^**2**^	***p***
			***n***	%	***n***	%	***n***	%		
Satisfaction with socio-physical environment	Trust in neighbors	No	911	6.6	1754	12.6	1380	9.9	13.99	.001
		Yes	1971	14.2	4550	32.8	3309	23.9		
	Help from neighbors	No	1563	11.0	2886	20.3	2447	17.2	69.97	< .001
		Yes	1392	9.8	3561	25.1	2360	16.6		
	Neighborhood safety level	No	520	3.6	987	6.9	805	5.6	9.49	.009
		Yes	2467	17.1	5557	38.6	4063	28.2		
	Natural environment	No	629	4.3	1391	9.5	1048	7.2	0.26	.876
		Yes	2400	16.5	5217	35.8	3884	26.7		
	Life environment	No	377	2.6	729	5.0	561	3.8	3.88	.144
		Yes	2663	18.3	5877	40.3	4381	30.0		
	Condition of public transportation	No	497	3.4	970	6.7	681	4.7	9.84	.007
		Yes	2536	17.4	5635	38.7	4245	29.1		
	Condition of medical facilities	No	475	3.3	864	5.9	661	4.5	12.05	.002
		Yes	2558	17.6	5726	39.4	4262	29.3		
Social support networks	Contact with family	Less than once a month	612	4.1	833	5.7	1068	7.3	229.51	< .001
		Once a month	327	2.2	848	5.8	701	4.8		
		2–3 times a month	397	2.7	961	6.5	714	4.9		
		Once a week	464	3.1	1112	7.6	633	4.3		
		2–3 times a week	467	3.2	1026	7.0	646	4.4		
		≥ 4 times a week	788	5.4	1864	12.7	1220	8.3		
	Contact with neighbors	Less than once a month	895	6.1	1960	13.4	1586	10.9	67.38	< .001
		Once a month	165	1.1	446	3.1	333	2.3		
		2–3 times a month	157	1.1	436	3.0	325	2.2		
		Once a week	176	1.2	526	3.6	404	2.8		
		2–3 times a week	404	2.8	909	6.2	637	4.4		
		≥ 4 times a week	1243	8.5	2327	15.9	1667	11.4		
	Contact with friends	Less than once a month	967	6.6	1691	11.5	1413	9.6	109.40	< .001
		Once a month	358	2.5	1057	7.2	724	4.9		
		2–3 times a month	264	1.8	795	5.4	604	4.1		
		Once a week	252	1.7	652	4.5	472	3.2		
		2–3 times a week	395	2.7	918	6.3	579	4.0		
		≥ 4 times a week	818	5.6	1524	10.4	1174	8.0		
Participation in social activities	Religious activities	No	1757	12.0	4206	28.6	2962	20.2	35.85	< .001
		Yes	1302	8.9	2438	16.6	2021	13.7		
	Belonging to communities	No	1719	11.7	2557	17.4	2330	15.9	275.06	< .001
		Yes	1340	9.1	4087	27.8	2653	18.1		
	Leisure activities	No	2563	17.4	5005	34.1	4006	27.3	101.15	< .001
		Yes	496	3.4	1639	11.2	976	6.6		
	Charity activities	No	2904	19.8	6163	42.0	4679	31.8	17.75	< .001
		Yes	155	1.0	481	3.3	303	2.1		

^a^ No responses were excluded

### Logistic regression analyses

The results for the logistic regression of the general characteristics; depressive symptoms; present health status; satisfaction with socio-physical environment; social support networks; participation in social activities; and happiness of older adults living alone, those living with their spouse, and those living with their family are presented in Table 4. The model was constructed with happiness as the dependent variable; sociodemographic characteristics, depressive symptoms, present health status, satisfaction with socio-physical environment, social support networks, and participation in social activities were independent variables. Separate models were derived for each of the three living arrangements groups of older adults. The models for older adults living alone (− 2 Log L = 3100.816, chi-square = 643.169, *p* < .001), those living with their spouse (− 2 Log L = 6275.436, chi-square = 1140.529, *p* < .001), and those living with their family (− 2 Log L = 4914.185, chi-square = 842.456, *p* < .001) met the convergence criterion for logistic regression.

**Table 4:** Logistic Regression Model for Happiness Comparing Three Living Arrangements of Older Adults

Variables	Living alone		Living with their spouse		Living with their family	
	OR	95% CI	OR	95% CI	OR	95% CI
Gender	1.39	1.08–1.78	1.06	0.92–1.22	1.39	1.16–1.66
Age	1.01	1.00–1.03	1.02	1.01–1.03	1.02	1.01–1.03
Educational level						
None	0.64	0.41–1.00	0.62	0.45–0.87	0.46	0.33–0.65
Elementary	0.85	0.57–1.26	0.63	0.50–0.80	0.57	0.43–0.77
Middle	0.63	0.42–0.95	0.55	0.44–0.70	0.61	0.45–0.82
High	0.72	0.48–1.09	0.73	0.58–0.92	0.61	0.45–0.82
College or higher	referent		referent		referent	
Marital status						
Married	0.90	0.47–1.75	1.64	0.08–31.79	0.48	0.19–1.22
Divorced	1.05	0.59–1.87	0.91	0.04–21.87	0.29	0.11–0.78
Widowed	1.18	0.68–2.07	1.00	0.04–24.11	0.50	0.19–1.28
Never married	referent		referent		referent	
Employment status	0.74	0.59–0.92	0.90	0.78–1.04	0.95	0.80–1.13
Eligibility for basic livelihood rights						
Yes	0.68	0.53–0.88	1.00	0.74–1.34	0.83	0.61–1.13
In the past	0.82	0.40–1.68	1.23	0.51–2.93	0.88	0.50–1.56
No	referent		referent		referent	
Monthly income (10,000 won)^a^						
< 50	0.48	0.32–0.72	0.37	0.28–0.47	0.53	0.36–0.78
50–99	0.77	0.52–1.14	0.56	0.46–0.67	0.63	0.51–0.79
100–199	0.81	0.53–1.24	0.64	0.54–0.76	0.69	0.58–0.82
≥ 200	referent		referent		referent	
Depressive symptoms	0.87	0.85–0.90	0.87	0.85–0.89	0.87	0.85–0.89
Present health status	1.21	1.15–1.28	1.26	1.21–1.31	1.23	1.18–1.29
Trust in neighbors	1.45	1.16–1.82	1.32	1.13–1.54	1.19	1.01–1.42
Help from neighbors	0.88	0.71–1.09	1.10	0.95–1.27	1.11	0.95–1.31
Neighborhood safety level	1.24	0.96–1.60	1.04	0.87–1.26	0.94	0.77–1.16
Natural environment	1.05	0.83–1.34	1.02	0.86–1.20	1.32	1.09–1.59
Life environment	1.01	0.76–1.36	1.14	0.93–1.41	1.23	0.97–1.56
Condition of public transportation	1.01	0.77–1.32	1.24	1.03–1.50	1.22	0.97–1.53
Condition of medical facilities	1.16	0.88–1.53	1.04	0.85–1.27	0.92	0.73–1.16
Contact with family						
Less than once a month	0.92	0.70–1.21	0.99	0.81–1.22	0.80	0.65–0.99
Once a month	0.70	0.51–0.95	0.84	0.68–1.02	0.97	0.77–1.22
2–3 times a month	0.87	0.64–1.18	1.07	0.87–1.31	0.92	0.73–1.15
Once a week	0.79	0.60–1.05	1.17	0.97–1.42	1.06	0.83–1.35
2–3 times a week	0.75	0.57–0.99	1.03	0.84–1.25	0.97	0.77–1.24
≥ 4 times a week	referent		referent		referent	
Contact with neighbors						
Less than once a month	0.90	0.70–1.15	0.83	0.69–0.99	1.04	0.86–1.27
Once a month	0.93	0.62–1.38	0.81	0.63–1.05	1.35	1.00–1.83
2–3 times a month	1.38	0.89–2.13	1.13	0.86–1.48	1.62	1.18–2.22
Once a week	0.62	0.42–0.93	0.88	0.68–1.12	0.85	0.65–1.12
2–3 times a week	1.01	0.76–1.34	1.12	0.91–1.37	1.07	0.85–1.35
≥ 4 times a week	referent		referent		referent	
Contact with friends						
Less than once a month	0.81	0.63–1.04	0.84	0.69–1.02	0.90	0.73–1.12
Once a month	0.93	0.68–1.26	0.69	0.56–0.84	0.91	0.72–1.15
2–3 times a month	0.90	0.64–1.27	0.80	0.64–1.01	1.10	0.85–1.41
Once a week	1.18	0.83–1.69	0.81	0.63–1.04	1.32	0.99–1.75
2–3 times a week	1.44	1.05–1.96	0.92	0.73–1.15	1.19	0.92–1.54
≥ 4 times a week	referent		referent		referent	
Religious activities	1.49	1.24–1.79	1.22	1.07–1.40	1.31	1.13–1.51
Belonging to communities	1.25	1.03–1.52	1.20	1.05–1.39	1.08	0.92–1.27
Leisure activities	1.38	1.05–1.80	1.47	1.24–1.75	1.37	1.12–1.68
Charity activities	1.08	0.70–1.67	1.22	0.91–1.62	1.20	0.86–1.68
Constant	0.04		0.02		0.03	

^a^ 1 USD = approximately 1200 Korean won. OR = odds ratio. CI = confidence interval

Significant factors influencing happiness in the three living arrangement groups were as follows. Among older adults living alone, females were 1.39 times more likely to be happy than were males (odds ratio (OR) = 1.39, 95% confidence interval (CI) = 1.08–1.78). As compared to participants with a college graduate degree or higher, happiness was 37% lower in middle school graduates (OR = 0.63, 95% CI = 0.42–0.95). Further, happiness was 26% lower in older adults who were employed (OR = 0.74, 95% CI = 0.59–0.92) than in unemployed older adults living alone, while happiness was 32% lower in those who were eligible for basic livelihood rights (OR = 0.68, 95% CI = 0.53–0.88) than in those not eligible for basic livelihood rights. Happiness was 52% lower in those with a monthly income of less than 500,000 won (OR = 0.48, 95% CI = 0.32–0.72) than for those with a monthly income of over 2,000,000 Korean won. Further, happiness scores decreased with an increase in depressive symptoms (OR = 0.87, 95% CI = 0.85–0.90), and increased with an increase in the present health status level (OR = 1.21, 95% CI = 1.15–1.28). With reference to social support networks, those who trusted their neighbors were 1.45 times more likely to be happy than those who did not (OR = 1.45, 95% CI = 1.16–1.82). Further, happiness was lower in those who had contact with their family only once a month (OR = 0.70, 95% CI = 0.51–0.95) or two to three times a week (OR = 0.75, 95% CI = 0.57–0.99), respectively, than those who had contact with their family four or more times a week. Similarly, happiness was 38% lower in those who had contact with neighbors only once a week (OR = 0.62, 95% CI = 0.42–0.93) than those who had contact with neighbors four or more times a week. Happiness was 1.44 times higher in those who had contact with friends two to three times a week (OR = 1.44, 95% CI = 1.05–1.96) than those who had contact with friends four or more times a week. With reference to participation in social activities, those who participated regularly in religious activities were 1.49 times more likely to be happy than those who did not (OR = 1.49, 95% CI = 1.24–1.79). Further, those who participated regularly in belonging to communities were 1.25 times more likely to be happy than those who did not (OR = 1.25, 95% CI = 1.03–1.52), and those who participated regularly in leisure activities were 1.38 times more likely to be happy than those who did not (OR = 1.38, 95% CI = 1.05–1.80).

Among older adults living with their spouse, as age increased, the probability of being happy increased (OR = 1.02, 95% CI = 1.01–1.03). As compared to those who were college graduates or higher, all those with an educational level of below high school graduation were less likely to be happy (OR = 0.73, 95% CI = 0.58–0.92). As compared to those with a monthly income of over 2,000,000 Korean won, those with a monthly income of below 1,990,000 Korean won were less likely to be happy (OR = 0.64, 95% CI = 0.54–0.76). Further, while happiness decreased with an increase in depressive symptoms (OR = 0.87, 95% CI = 0.85–0.89), it increased with an increase in present health status level (OR = 1.26, 95% CI = 1.21–1.31). Those who trusted their neighbors were 1.32 times more likely to be happy than those who did not (OR = 1.32, 95% CI = 1.13–1.54). Additionally, those who were satisfied with condition of public transportation were 1.24 times more likely to be happy than those who were not (OR = 1.24, 95% CI = 1.03–1.50). As compared to those who had contact with neighbors four or more times a week, happiness was 17% lower in those who had contact with neighbors less than once a month (OR = 0.83, 95% CI = 0.69–0.99). Similarly, as compared to those who had contact with friends four or more times a week, happiness was 31% lower in those who had contact with friends only once a month (OR = 0.69, 95% CI = 0.56–0.84). Regarding participation in social activities, those who participated regularly in religious activities were 1.22 times more likely to be happy than those who did not (OR = 1.22, 95% CI = 1.07–1.40), while those who participated regularly in belonging to communities were 1.20 times more likely to be happy than those who did not (OR = 1.20, 95% CI = 1.05–1.39). Additionally, those who participated regularly in leisure activities were 1.47 times more likely to be happy than those who did not (OR = 1.47, 95% CI = 1.24–1.75).

Among older adults living with their family, females were 1.39 times more likely to be happy than were males (OR = 1.39, 95% CI = 1.16–1.66). Further, the probability of being happy increased with an increase in age (OR = 1.02, 95% CI = 1.01–1.03). As compared to those with a college graduate degree or higher, those with an educational level of high school graduation or lower were less likely to be happy (OR = 0.61, 95% CI = 0.45–0.82). Further, as compared to older adults who had never married and lived with their family, happiness was 71% lower in those who were divorced (OR = 0.29, 95% CI = 0.11–0.78). As compared to those with a monthly income of over 2,000,000 Korean won, those with a monthly income of below 1,990,000 Korean won were less likely to be happy (OR = 0.69, 95% CI = 0.58–0.82). While the happiness score decreased with an increase in depressive symptoms (OR = 0.87, 95% CI = 0.85–0.89), it increased with an increase in present health status level (OR = 1.23, 95% CI = 1.18–1.29). Further, those who trusted their neighbors were 1.19 times more likely to be happy than those who did not (OR = 1.19, 95% CI = 1.01–1.42). Additionally, those who were satisfied with their natural environment were 1.32 times more likely to be happy than those who were not (OR = 1.32, 95% CI = 1.09–1.59). As compared to those who had contact with their family four or more times a week, happiness was 20% lower in those who had contact with their family less than once a month (OR = 0.80, 95% CI = 0.65–0.99). As compared to those who had contact with neighbors four or more times a week, happiness was 1.62 times higher in those who had contact with neighbors two to three times a week (OR = 1.62, 95% CI = 1.18–2.22). With reference to participation in social activities, those who participated regularly in religious activities were 1.31 times more likely to be happy than those who did not (OR = 1.31, 95% CI = 1.13–1.51). Similarly, those who participated regularly in leisure activities were 1.37 times more likely to be happy than those who did not (OR = 1.37, 95% CI = 1.12–1.68).

## Discussion

This study investigated the associations between depressive symptoms, present health status, satisfaction with socio-physical environment, social support networks, and participation in social activities with happiness among older adults living alone, those living with their spouse, and those living with their family.

### Associations between subject characteristics, depression, and health status with happiness

In this study, educational level, monthly income, depressive symptoms, and present health status were the common factors associated with happiness in the three groups based on living arrangements. These results were consistent with Kim’s [22] finding that education and household income affect the quality of life of older adults. According to Van Leeuwen et al. [14], financial resources affect older adults’ quality of life, feelings of independence, and access to a comfortable life. Additionally, the present findings were consistent with those of previous studies [7, 14, 22–24] that reported that depression and health status were closely related to happiness. Further, Kim, Song, Kim, and Park [23] reported that depressive symptoms were powerful predictors of happiness in older women living alone. Similarly, Sakamoto et al. [24] found that, among older adults living alone, a higher level of depression was significantly associated with a low score on subjective happiness. In the present study, among older adults living alone and those living with their family, females were likely to be happier when compared to males. According to Tomioka, Kurumatani, and Hosoi [25], although different social participation programs were implemented for older males and females, most programs catered to females. Therefore, programs targeting older males need to be developed to improve their happiness levels. The present study also found that, among older adults living with their spouse and those living with their family, happiness increased with age. Specifically, in this study, older adults living with their spouse had the highest happiness score, while those living alone had the lowest happiness score. Further, older adults living alone had the highest depression score and the lowest health status score. It was also found that divorced participants were less likely to be happy than participants who had never married. On the contrary, older adults living with their spouse had the lowest level of depression and the best overall present health status. According to Baumann et al. [15], older adults’ life satisfaction decreased slightly with age, which was not consistent with the present results. However, this result was consistent with the findings of them [15], which suggested that life satisfaction was positively related to living with a spouse rather than living alone. Further, the present results were similar to the findings of Robins et al. [26], who reported that happiness was significantly associated with lower social isolation, and that participants living with their spouse exhibited better general health, higher levels of household-based physical activity, and lower levels of depression as compared to their counterparts. Grundy and Murphy [8] also reported lower levels of happiness among those with poorer health and fewer social resources. Together, these studies suggest that having a spouse could be one of the most important factors influencing happiness in old age.

In the present study, employment status and eligibility of basic livelihood rights were not significantly correlated with happiness in older adults living with their spouse and in those living with their family. However, older adults living alone had low levels of happiness when they were employed or when they were eligible for basic livelihood rights. These results were consistent with the findings of Baumann et al. [15], who reported that retired older adults had higher life satisfaction as compared to employed older adults. Dingemans and Henkens [27] found that older adults with a poor socio-economic background have limited career choices, and therefore are forced to work in unfavorable conditions. Most retired older adults have a low income because they engage in part-time work, or their job requires low physical and mental effort. Their low income renders them susceptible to poverty, especially when they live alone. The present study found that, among those with a monthly income of more than 2,000,000 Korean won, the proportion of older adults living alone was far lower than that of older adults living with their spouse and those living with their family. These results suggest that it may be difficult for older adults living alone to derive satisfaction and happiness from their work.

### Association of Socio-Physical Environment with happiness

In this study, the common aspect of socio-physical environment that was associated with happiness in all three living arrangement groups was trust in neighbors. This was similar to a previous study [28] that found that depression was lower in older adults who had good ties with their neighbors. Similarly, Wu and Chan [29] found that living in a public apartment and daily participation in public neighborhood events reduced the risk of isolation in older adults. Additionally, Lee et al. [13] reported that residents in a community with strong mutual trust had a higher quality of life than did those without trust. They added that this affected health-related quality of life through the creation of a mutually supportive environment, such as belonging to communities, mutual exchange, attachment to neighbors, and so on. Furthermore, the present results revealed that condition of public transportation was significantly associated with the happiness of older adults living with their spouse. The prior studies could not be found on the basis of public transport status affecting happiness of older adults living with their spouse. However, the present results showed that older adults living with their spouse had the highest participation rate in social activities compared to the other two groups. In order to facilitate participation in social activities, it may be considered that public transportation was considered important. Therefore, the present results showed that the natural environment and condition of public transportation had a significant effect on happiness. This finding was similar to that of a previous study [30], which reported that the safer the living environment and the lower the car or subway traffic obstacles, the better was the subjective and mental health of residents. It is important to focus on improving such aspects of the physical environment to improve health and happiness of older adults.

### Association of Social Support Networks with happiness

In this study, the common aspect of social support networks that was associated with happiness in all three living arrangement groups was contact with neighbors. However, the nature of this association differed between the three groups. Specifically, happiness among older adults living alone and those living with their spouse increased in proportion to their contact with neighbors. In contrast, older adults living with their family were happier if they had optimal rather than excessive contact with their neighbors. This finding suggests that, in older adults living with their family, contact with neighbors or friends did not seem to be significantly associated with happiness. The older adults living with their family were more dependent and focused on the family, such as children [14]. Therefore, this study showed that neighbors are an important support system for older adults living alone and for those living with their spouse. These results are consistent with the finding of Van Leeuwen et al. [14], who reported that the quality of life of older adults was related to their relationships with neighbors. They found that older adults with friendly neighbors and a sense of familiarity in their neighborhood had a higher sense of security [14]. Similarly, Wu and Chan [29] found that if older adults living alone in an apartment interacted with other residents and continued to participate in community events, they demonstrated a decrease in loneliness.

Regarding the contact with family and friends, the present findings differed across the three living arrangement groups. Older adults living alone and those living with their family exhibited an increase in happiness with an increase in the contact with their family. In contrast, older adults living with their spouse exhibited higher happiness when their contact with friends was higher. Specifically, many older adults living alone reported little participation in social activities, and their children were the major support source [31]. These findings were consistent with those of the present study. Chiang and Lee [3] found that family relations were positively correlated with happiness, sense of coherence, and perceived health in older adults. Bai, Yang, and Knapp [6] reported that sense of loneliness mediated the effects of support from family, friends, and others on life satisfaction. While older adults living with their family were dependent on their children for care, older adult couples (i.e., those living only with their spouse) were characterized by their independence in their relationships with their children and by their more active lives [14]. Grundy and Murphy [8] reported that widows living with a child were happier than those living without a child (generally alone). Therefore, more efforts are needed to improve the social support network of older adults living alone as compared to those living with their spouse or family.

In the present study, in older adults living alone, family and neighborhood contact were significantly correlated with happiness, which was consistent with the findings of previous studies [29, 31]. Further, in this study, the average age of older adults living alone was the highest among the three groups; their spouses were likely to have died, leaving them alone as they aged. With aging, physical functions weaken and social networks shrink. Loneliness, usually because of bereavement or moving into a new community, has a strong negative impact on older adults’ quality of life [14]. Losing connections with friends and family members was another difficult aspect for older adults [14]. Evidently, those with poor family relationships may need more social welfare services. Social support and mutual supportive communities have been found to have a significant effect on the self-efficacy and health-related quality of life of older adults [13]. Social relations are strongly related with life satisfaction [7]. Meanwhile, Djundeva, Dykstra, and Fokkema [17] indicated that older adults with “restricted” networks tend to have the poorest well-being, but those with “diverse” networks have even better well-being than co-residing older adults. Therefore, to improve older adults’ happiness levels, it is necessary to provide various social support networks considering their living arrangements.

### Association of Social Activities with happiness

In this study, the common aspects of participation in social activities that were associated with happiness in all three living arrangement groups were religious activities, and leisure activities. Religious meetings and group activities have a significant effect on the life satisfaction of older adults living alone [16]. The present results were similar to the findings of Ofstedal et al. [32], who reported that older adults participating in religious services exhibited better health expectancy. Being religious or spiritual can support older adults’ acceptance of disability or psychological distress, coping with changes, and satisfaction with life [14]. Volunteering and taking part in religious activities and practices, such as going to church, were described as ways to stay socially active and involved [14]. Ofstedal et al. [32] added that attending religious services had a strong and consistent association with life and health expectancy.

The present results also indicated that older adults’ participation in leisure activities was positively associated with happiness. This finding is consistent with the results of a previous study [33], which found that older adults experienced social satisfaction and prevented social isolation by participating in various group activities. Further, the number of friends in their social network had a significant positive effect on their life satisfaction [33]. In a previous study [34], older adults’ physical activity level was positively associated with their psychological well-being. Wu and Chan [29] stated that, in older adults living alone in large cities, contact with friends was more effective in reducing loneliness than was contact with relatives who had no ties with them. However, if older adults have limited physical functioning, bonding with adult children has been found to be very important [29]. Therefore, if older adults living alone have no physical limitations and are healthy, socializing with friends and participating in leisure activities could help improve their subjective health and happiness. The present study also found that most older adults living alone had contact with friends two or three times per week, and that the more social activities they had, the higher was their happiness. This seems to favor the idea of being in contact with friends based on activities with a purpose rather than simply meeting them without any purpose. This finding suggests that experiencing healthy social exchanges with individuals who have experienced similar life processes could improve the quality of life of older adults. A previous study [33] reported that, in older adults living alone, participation in productive leisure activities, such as exercise, volunteer work, and travel, had a positive effect on their physical and mental health. Older adults who participated in various programs at senior welfare centers reported that their happiness improved, and depression decreased significantly [34, 35]. Similarly, De Koning, Richards, and Stathi [36] found that volunteering, accompanying others, and participating in sports/exercise were associated with lower social isolation in older adults. Further, Tomioka, Kurumatani, and Hosoi [25] found that social groups were the best form of social participation for community-dwelling older adults because they helped maintain their cognitive functioning abilities. Older adults have reported to have a negative perception of aging, and a strong desire to participate in health-related leisure activities [36]. In Together, these findings suggest that continued provision of and participation in social activities for older adults, especially for those living alone, may reduce depression and improve happiness. Therefore, governments and communities should continue to develop and provide social and leisure programs for older adults, especially for those living alone.

The interesting differences observed between the three groups have been summarized below.

First, trust in neighbors was found to be a significant influence factor of happiness in all three groups. While the physical environment was significantly associated with happiness among older adults living with their spouse and those living with their family, it was not so for older adults living alone. Second, with reference to social support networks, while contact with family and neighbors was significantly associated with happiness among older adults living alone, contact with neighbors and friends was an important factor for older adults living with their spouse, and contact with family was significant for older adults living with their family. Third, while social activity participation was significantly correlated with happiness in all three groups, this association was not observed with reference to participation in charity activities. Considering these characteristics, it is suggested that the elderly’s happiness promotion program needs to consider their living arrangements.

### Limitations

This study had several limitations. All the secondary data collected were cross-sectional, making it difficult to make causal inferences. Further, those who were depressed may have been more likely to live alone, and those living alone may be more likely to have a lower income as compared to those living with their families. In old age, income has a significant impact on quality of life [3]. Attempts to generalize the results of this study, which were obtained using secondary data originally collected for another purpose, must be undertaken with caution. The data acquired had inherent limitations. For example, we did not have information on whether participants had a history of major depression. The data used in this study were collected using a self-report questionnaire. Thus, the possibility of response bias cannot be eliminated. Finally, the data were obtained through the 2017 Korean Community Health Survey organized by the KDCA. While the data were collected simultaneously across the country, and data collectors had received adequate information about the survey in advance, there may have been individual differences in how they collected the data.

### Implications for further research

There is a need for longitudinal studies that consider participants’ characteristics, for example, severity of depression or other diseases. Additionally, we suggest the need for intervention studies that examine the mediating effect of happiness improvement programs using the factors associated with happiness in older adults identified in this study.

## Conclusions

This study aimed to compare the associations of depressive symptoms, present health status, satisfaction with socio-physical environment, social support networks, and participation in social activities with happiness in older adults living alone, with their spouse, and with their family. The group-wise results are summarized as follows.

Among older adults living alone, female gender; higher educational level; economic ability to survive without working; absence of depression; good health status; trusted neighbors; more frequent contact with family and neighbors; contact with friends two or three times a week; and regular participation in religious, belonging to communities, and leisure activities were associated with a higher likelihood of happiness. Among older adults living with their spouse, older age; higher educational level; income; absence of depression; good health status; convenience in using public transportation; more frequent contact with trusted neighbors and friends; and regular participation in religious, belonging to communities, and leisure activities were associated with a higher likelihood of happiness. Among older adults living with their family, female gender; older age; higher educational level; never having been divorced; income; absence of depression, good health status, more frequent contact with family, contact with trusted neighbors two or three times a week, and regular participation in religious and leisure activities were associated with a higher likelihood of happiness.

Together, the present findings suggest that the family is an essential support system for older adults. It was identified that factors associated with older adults’ happiness differed according to their living arrangements. Therefore, attempts to ensure the happiness of older adults must adequately account for their circumstances, including living arrangements. Governments and communities should improve the socio-physical environment for older adults and should continue to develop and provide social activity programs to improve their health and happiness considering various individual characteristics according to living arrangements.

## Data Availability

The data that support the findings of this study are available from the corresponding author upon reasonable request.
